# Comparative pharmacokinetics of free doxorubicin and a liposomal formulation in cats following intravenous administration

**DOI:** 10.3389/fvets.2024.1353775

**Published:** 2024-01-17

**Authors:** Yu Liu, Sumeng Chen, Zeyu Wen, Jinyan Meng, Yuxin Yang, Yang Zhang, Jianzhong Wang, Xingyuan Cao

**Affiliations:** ^1^Department of Veterinary Pharmacology and Toxicology, College of Veterinary Medicine, China Agricultural University, Beijing, China; ^2^Shanxi Key Laboratory for Modernization of TCVM, College of Veterinary Medicine, Shanxi Agricultural University, Jinzhong, China; ^3^Key Laboratory of Detection for Veterinary Drug Residue and Illegal Additive, Ministry of Agriculture and Rural Affairs, Beijing, China

**Keywords:** doxorubicin, doxorubicinol, comparative pharmacokinetics, feline, cancer

## Abstract

Doxorubicin, a potent chemotherapeutic agent used extensively in cancer treatment, displays complex pharmacokinetic behavior, especially across various formulations. With a rising incidence of cancer cases in cats, understanding the drug’s pharmacokinetics in feline subjects remains a critical yet unexplored area. Hence, this study investigated the pharmacokinetic profile of doxorubicin after slow intravenous administration of doxorubicin hydrochloride (DOX·HCl) or doxorubicin hydrochloride pegylated liposome (DOX·HCl-PLI) in twelve cats at a single dose of 20 mg/m^2^. Blood samples collected at pretreatment time (0 h) and over 192 h were analyzed using ultra-performance liquid chromatography-mass spectrometry (UPLC-MS/MS). The obtained pharmacokinetic parameters of doxorubicin revealed significant differences between the two formulations and were as follows: elimination half-life (T_1/2λz_) of 5.00 ± 3.20 h (DOX·HCl) and 17.62 ± 8.13 h (DOX·HCl-PLI), area under the concentration/time curve from 0 to last point (AUC_last_) of 0.67 ± 0.12 μg hr./mL (DOX·HCl) and 783.09 ± 267.29 μg hr./mL (DOX·HCl-PLI), and total body clearance (CL__obs_) of 27098.58 ± 5205.19 mL/h/m^2^ (DOX·HCl) and 28.65 ± 11.09 mL/h/m^2^ (DOX·HCl-PLI). Additionally, differences were also detected in the apparent volume of distribution (Vz__obs_) with 178.56 ± 71.89 L/m^2^ (DOX·HCl) and 0.64 ± 0.20 L/m^2^ (DOX·HCl-PLI), and the maximum plasma concentration (C_max_) with 2.25 ± 0.30 μg/mL (DOX·HCl) and 24.02 ± 5.45 μg/mL (DOX·HCl-PLI). Notably, low concentration of doxorubicinol, the metabolite of doxorubicin, was detected in plasma after administration of DOX·HCl, with even less present when DOX·HCl-PLI was administered. This investigation provides valuable insights into the distinct pharmacokinetic behaviors of DOX·HCl and DOX·HCl-PLI in cats, contributing essential groundwork for future studies and potential clinical applications in feline oncology.

## Introduction

Doxorubicin, widely used in the treatment of breast and ovarian cancer, is an anthracycline broad-spectrum antitumor antibiotic that inhibits DNA, RNA, and protein synthesis across cells in various growth cycles (1, 2). Currently, there are two Doxorubicin formulations authorized for clinical use in human treatments: hydrochloride doxorubicin and hydrochloride liposome doxorubicin (3). Although doxorubicin has significant antitumor efficacy, clinical studies have reported its many adverse effects (4–6). For instance, research has shown that doxorubicin and its metabolite, doxorubicinol, when combined elicit strong toxic side effects in humans and other species (7, 8). Investigations into a liposomal form of doxorubicin have revealed its capacity to alter the pharmacokinetic profile of the drug *in vivo*, consequently enhancing the drug’s anti-tumor efficacy (9–13).

Feline mammary carcinoma remains as one of the most common tumors in cat, showing high malignancy and metastasis rate, showing clinicopathological, epidemiological and histological features, and molecular classification resemble those found in human cancer (14–16). In recent years, several studies have gradually described the doxorubicin efficacy in the treatment of clinical tumors in cats across a variety of feline tumors (17–20). Specifically, one report detailed the use of debulking surgery and adjuvant doxorubicin chemotherapy in the treatment of mesenteric hemangiosarcoma, resulting in prolonged survival in the cat (18). However, other reports have expressed concerns regarding the drug’s significant toxicity in cats, leading to renal injury, myelosuppression, anorexia, and weight loss (21). A deep understanding is needed to unveil therapeutic options aimed at improving the cat’s clinical outcome. Such studies are limited by a lack of feline cell lines available for cytotoxicity assays (22). Moreover, when comparing the two formulations of doxorubicin, the physical properties of the liposome play a major role in altering the drug’s pharmacokinetics, a factor intricately tied to both efficacy and adverse reactions (23). Despite this, only a few studies have characterized the pharmacokinetic profile of doxorubicin in cats. Assessing the pharmacokinetic profile of doxorubicin and doxorubicinol in target species stands as a crucial step for comprehending efficacy and managing potential adverse effects in future clinical treatments. Therefore, the main purpose of the present study was to characterize the pharmacokinetics of doxorubicin and its metabolite, doxorubicinol, from two distinct doxorubicin formulations in cats.

## Materials and methods

### Materials

Doxorubicin hydrochloride standard product (purity = 98.0%), doxorubicinol standard product (purity = 96.4%), and daunorubicin hydrochloride standard product (Internal standard, purity = 98.8%) were provided from TLC Pharmaceutical Standers. Doxorubicin hydrochloride for injection (DOX·HCl, 10 mg) was obtained from ShanXi Pude Pharmaceutical Co., Ltd., and doxorubicin hydrochloride pegylated liposome injection (DOX·HCl-PLI, 20 mg:10 mL) was supplied by CSPC Pharmaceutical Group Ouyi Pharmaceutical Co., Ltd. All other chemicals and reagents utilized were of chromatographic grade and obtained from commercial suppliers (Fisher Scientific).

### Experiment design

Twelve domesticated Chinese pastoral cats (six females and six males; 3–3.6 kg; age range of 2–3 years) obtained from the Experimental Animal Center of China Agricultural University were used in this study. Prior to the study, cats were verified to be healthy based on physical examination. All procedures were reviewed and approved by the Institutional Animal Care and Use Committee of the China Agricultural University (NO.13303-21-E-001).

The preset experiment was conducted in a single-dose and parallel-dose design. Twelve cats were randomly allocated to two equally sized treatment groups. Group I received DOX·HCl at a dose of 20 mg/m^2^ body surface, while group II received DOX·HCl-PLI at the same dose. Similarly to clinical practice, both formulations were administered via slow intravenous (i.v.) infusion (administered over 10 minutes) after dilution with 0.9% NaCl, maintaining an injection rate of approximately 10 μL/s. The cats fasted for 16 h before and 8 h following drug administration. Each cat was weighed immediately prior to drug administration on the first day of treatment.

Blood samples of 0.5 mL were collected into heparinized tubes via the forelimb veins at 0 (pretreatment) and 0.033, 0.083, 0.167, 0.25, 0.5, 0.75, 1, 1.5, 3, 6, 12, 24, 48, 72, 96, 120, 144, 168 and 192 h after starting administration. Afterward, blood samples were centrifuged for 10 min at 4000 rpm, and plasma samples were stored in −20°C until analysis.

### Samples analysis

Plasma concentrations of doxorubicin and doxorubicinol were analyzed using a validated ultra-performance liquid chromatography-mass spectrometry (UPLC-MS/MS) method as previously reported (7, 24). The lower limit of quantification (LLOQ) for doxorubicin and doxorubicinol in plasma were 10 ng/mL and 2 ng/mL, respectively. Both inter- and intra-assay coefficients of variation remained below 15%. Mean recoveries of doxorubicin ranged from −14.51 to 10.00%, while those of doxorubicinol ranged between −14.00 to 10.63%. Calibration curves of doxorubicin and doxorubicinol exhibited satisfactory linearity within the concentration range of 10 to 2000 ng/mL (*r*^2^ > 0.99) and 2 to 400 ng/mL (*r*^2^ > 0.99), respectively.

### Data analysis

Pharmacokinetic parameters were determined from the Noncompartmental Analysis Model 200–202 (Linear Trapezoidal Linear Interpolation Method, Uniform Weighting) in WinNonlin™ software (WinNonlin 8.3, Certara United States). To evaluate significant differences, a *T*-test via SPSS Statistics 20.0 (International Business Machines, Armonk, NY, United States) was employed between study groups following logarithmic transformation in elimination half-life (T_1/2λz_), area under concentration/time curve from 0 to last point (AUC_last_), total body clearance (CL__obs_), apparent volume of distribution (Vz__obs_), and maximum plasma concentration (C_max_). Statistically significant differences were considered for *p*-values (p) below 0.01 and all data are expressed as mean ± standard deviation (SD).

## Results

**Figure 1:**
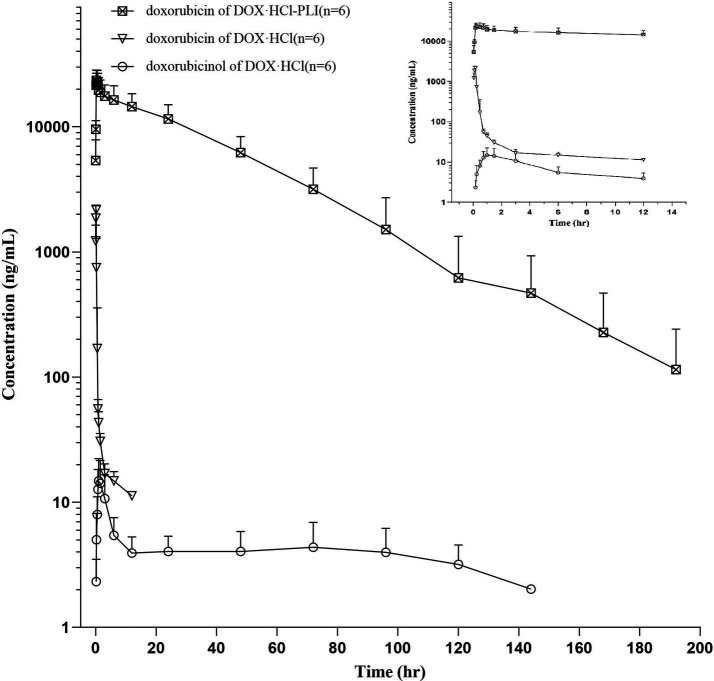
Plasma concentration/time curves of doxorubicin and doxorubicinol in cats (*n* = 6) following a slow i.v. injection of a single dose (20 mg/m^2^) of DOX·HCl or DOX·HCl-PLI, respectively. A close-up view is shown for the first 12 h post drug administration.

The plasma concentration/time curves of doxorubicin for both DOX·HCl and DOX·HCl-PLI groups are displayed in Figure 1. Additionally, the curve for doxorubicinol is presented for the DOX·HCl group, while the curve for the DOX·HCl-PLI group is not shown due to limited measured time points post-administration. The results demonstrated that the pharmacokinetic profile of doxorubicin after administration of the two formulations was significantly different.

The pharmacokinetic parameters for the two doxorubicin formulations are presented in Table 1. The results showed significant differences between the two groups, DOX·HCl and DOX·HCl-PLI, in the T_1/2λz_, AUC_last_, CL__obs_, Vz__obs_, and C_max_ values (*p* < 0.01). Given the low concentration of metabolite doxorubicinol and the limited number of measured time points, the pharmacokinetic parameters of doxorubicinol could not be accurately obtained based on the present dose of doxorubicin. No adverse effects were observed in any cat.

**Table 1:** Plasma pharmacokinetic parameters (arithmetic mean ± SD) after i.v. infusion administration of a single dose (20 mg/m^2^) of the two doxorubicin formulations to cats (*n* = 6), DOX·HCl and DOX·HCl-PLI.

Parameters	DOX·HCl (*n* = 6)	DOX·HCl-PLI (*n* = 6)
λ_z_ (1/h)	0.17 ± 0.07	0.05 ± 0.03
T_1/2λz_ (hr)	5.00 ± 3.20^**^	17.62 ± 8.13^**^
MRT_last_ (hr)	0.83 ± 0.36	32.72 ± 6.17
T_max_ (hr)	0.14 ± 0.04	0.29 ± 0.10
AUC_INF_obs_ (μg·hr./mL)	0.76 ± 0.15	785.83 ± 270.56
AUC_last_ (μg·hr./ mL)	0.67 ± 0.12^**^	783.09 ± 267.29^**^
CL__obs_ (mL/h/m^2^)	27098.58 ± 5205.19^**^	28.65 ± 11.09^**^
Vz__obs_ (L/m^2^)	178.56 ± 71.89^**^	0.64 ± 0.20^**^
C_max_ (μg/ mL)	2.25 ± 0.30^**^	24.02 ± 5.45^**^

Pharmacokinetic parameters were calculated using non-compartmental Analysis Model 200–202 in Winnonlin ™ software (WinNonlin 8.3, Certara USA); λz, the elimination rate constant; T1/2λz, elimination half-life; MRTlast, mean residence time; Tmax, time to reach the observed maximum (peak) concentration at steady-state; AUCINF_obs, area under concentration/time curve from 0 to infinity; AUClast, area under concentration versus time curve from 0 to last point; CL_obs, total body clearance; Vz_obs, the apparent volume of distribution; Cmax, maximum plasma concentration. ^**^Significant differences (*p* < 0.01), DOX·HCl group compared with DOX·HCl-PLI group.

## Discussion

Doxorubicin has shown significant therapeutic efficacy in many cancer types, being considered one of the most potent approved chemotherapeutic drugs. Previous pharmacokinetic studies primarily focused on humans and other species (25); however, there has been a rising trend in reported clinical tumor cases among cats in recent years (18, 21). Thus, our study investigated the pharmacokinetics of two formulations of doxorubicin measuring the plasma concentrations of doxorubicin and its metabolite, doxorubicinol, in cats. Consistent with common findings, our results detected doxorubicin and doxorubicinol in plasma samples, with the parent drug predominantly present in the plasma after administration (26).

Our findings showed significant differences in the pharmacokinetic profiles of doxorubicin and doxorubixinol between DOX·HCl and DOX·HCl-PLI formulations. These results align with those of A. Gabixon et al., wherein the study of the doxorubicin liposome formulation reported a longer circulation time in plasma and a smaller apparent volume of distribution following i.v. administration compared to conventional doxorubicin (27). Moreover, our study indicated a longer elimination half-life after administration of DOX·HCl-PLI compared to DOX·HCl group. Of note, the same trait was also observed in other species, as shown by the elimination half-life of 27 ± 5 h after administration of liposomal doxorubicin in dogs (28). The presence of polyethylene glycol (PEG) molecules on the surface of liposomal doxorubicin contributes to its extended circulation times *in vivo*, exceeding 18 h in mice and 50 h in humans (29). The apparent difference in circulation time between the two formulations is probably attributed to the PEG coat that reduces their interaction with the mononuclear phagocyte system (MPS), thereby aiding in bypassing elimination in the liver (30). Additionally, the volume of distribution of DOX·HCl-PLI group was significantly smaller in comparison to the DOX·HCl group, indicating that the liposomal formulation is mostly confined within the intravascular compartment (2). Furthermore, the AUC_last_ (0.67 ± 0.12 μg·hr./mL) of DOX·HCl was lower than of the DOX·HCl-PLI (783.09 ± 267.29 μg·hr./mL) in cats.

These results suggest that DOX·HCl-PLI potentially enhances bioavailability and improves its potential for anti-tumor effects. In a study by A. Rahmab et al., the comparative pharmacokinetics of free doxorubicin and doxorubicin entrapped in cardiolipin liposomes were explored in rats administered at an i.v. dose of 6 mg/kg. The peak plasma concentration of free doxorubicin at 5 min was 1.7 μg/mL, while the cardiolipin liposomes formulation produced a peak plasma concentration of doxorubicin at 5 min of 20.9 μg/mL. The AUC for the free doxorubicin and the liposomal doxorubicin was 1.95 μg·hr./mL and 81.4 μg·hr./mL, respectively (31). Similarly, in mice, the AUC_last_ (72.98 ± 15.79 μg·hr./mL) of DOX·HCl was lower than of the DOX·HCl-PLI (499.61 ± 72.35·μg hr./mL) (32).

The present findings suggest that DOX·HCl exhibits a larger volume of distribution, faster clearance, and a shorter half-life in comparison to DOX·HCl-PLI.

Nevertheless, our study could detect that the concentration of doxorubicinol was low immediately after administration of DOX·HCl, with even less present when DOX·HCl-PLI was administered. These observations are in agreement with previous findings (29, 33) and indicate a notably reduced conversion of doxorubicin to doxorubicinol when doxorubicin is administrated in its liposomal form. The data in the present study could potentially serve as scientific evidence contributing to explaining the substantially reduced cardiotoxicity associated with liposomal doxorubicin.

In conclusion, this research represents an initial investigation into understanding the pharmacokinetics of two distinct formulations of doxorubicin in cats. While preliminary, the findings offer fundamental insights that could prove crucial for the practical and clinical administration of doxorubicin, particularly in treating cancer in feline subjects.

## Data availability statement

The original contributions presented in the study are included in the article/supplementary material, further inquiries can be directed to the corresponding authors.

## Ethics statement

The animal study was approved by Institutional Animal Care and Use Committee of the China Agricultural University (NO. 13303-21-E-001). The study was conducted in accordance with the local legislation and institutional requirements.

## Author contributions

YL: Writing – original draft, Formal analysis, Validation, Writing – review & editing. SC: Writing – review & editing, Formal analysis, Validation. ZW: Writing – review & editing, Validation. JM: Writing – review & editing, Data curation, Validation. YY: Writing – review & editing, Validation. YZ: Writing – review & editing, Validation. JW: Writing – review & editing. XC: Writing – review & editing.
